# Comparative study of single-center patients with thyroid metastases from colorectal cancer and previously reported cases in the literature

**DOI:** 10.1186/s12957-017-1140-5

**Published:** 2017-04-20

**Authors:** Adili Keranmu, Hongtu Zheng, Yuchen Wu, Jiang Zhao, Xiaolin Xu, Fangqi Liu, Sanjun Cai, Yu Wang, Ye Xu

**Affiliations:** 1 Department of Colorectal Surgery, Fudan University Shanghai Cancer Center, No. 270, Dong An Road, Shanghai, 200032 China; 2 Department of Pathology, Fudan University, No. 130, Dong An Road, Shanghai, 200032 China; 3 Department of Head and Neck Surgery, Fudan University Shanghai Cancer Center, No. 270, Dong An Road, Shanghai, 200032 China; 4 Department of Oncology, Shanghai Medical College, Fudan University, No. 130, Dong An Road, Shanghai, 200032 China

**Keywords:** Colon cancer, Rectal cancer, Thyroid metastasis

## Abstract

**Background:**

Thyroid metastases from colorectal cancer (CRC) are rare, both in clinical practice and in the literature; hence, their diagnosis, appropriate treatment, and prognostic factors require further investigations.

**Methods:**

A retrospective analysis was performed for four cases of thyroid metastases from CRC, treated in our center between January 2005 and December 2015, and the relevant literature was searched using PubMed, resulting in the identification of 17 patients with detailed information available. The clinical data and follow-up information of our patients and the previously reported cases were collected and compared.

**Results:**

The median age of the 21 patients was 59 years (44.5 and 66 years for our patients and the previously reported cases, respectively). Fifteen (71.4%) primary tumors were distributed throughout the distal colon or rectum (75% [3/4] in our center and 70.5% [12/17] in the previously reported cases). According to our analysis, we found that 81.0% of patients (17/21) showed concomitant lung metastasis. Among them, all four patients in our center showed lung metastasis, and 75% (3/4) developed thyroid metastases after the lung metastasis. In the previously reported cases, the corresponding proportions were 76.5 and 76.5% (13/17) of patients, respectively. The median time after primary tumor diagnosis to thyroid metastasis development was 28 months (26 months in our center and 35 months in the previously reported cases). One patient with advanced CRC in our center died 5 months after the thyroid metastasis was identified, while the remaining three patients are currently alive (longest follow-up time, 27 months). The median survival time after thyroid metastasis during 3 years of follow-up of the previously reported 17 patients was 12 months. There was no difference in the overall survival between patients treated non-surgically (8/21) and patients undergoing thyroidectomy alone or thyroidectomy with adjuvant therapy (13/21) (*p =* 0.388). In addition, we found that the overall survival of the patients whose other metastases were treated with radical treatment was superior to that in those treated with palliative treatment (*p =* 0.022).

**Conclusions:**

Thyroid metastases from CRC are rare in clinical practice and are a manifestation of advanced CRC. The prognosis of patients with thyroid metastases from CRC is related to various factors, including the grade of malignancy of the primary lesion, the presence of other metastases, and whether the metastases are timely diagnosed and a radical treatment strategy is employed.

## Background

Thyroid metastasis of malignant tumors is observed in 1.9–9.5% of histologically examined autopsy cases [1]. Nevertheless, in clinical practice, metastases to the thyroid from non-thyroid malignancies remain a rare occurrence, comprising only 1.4–3% of all thyroid neoplasms [2]. Moreover, most patients are asymptomatic early in the disease course and thyroid metastases are generally discovered during routine follow-up examinations. However, currently, routine follow-up examinations of thyroid metastases from colorectal cancer (CRC), including thyroid ultrasonography and computed tomography (CT), are not commonly performed, and this may lead to misdiagnosis or delayed diagnosis of thyroid metastases from CRC.

Here, we describe four cases of thyroid metastases from CRC treated at our center (Table 1) and compare these to 17 previously reported cases in the literature (Table 2).

**Table 1:** Four cases of thyroid metastases from CRC, treated in our center between January 2005 and December 2015

Case	Age (year)/sex	Primary site	Histology	Treatment	Site of thyroid metastasis	Time from primary lesion diagnosis (months)	Clinical manifestation	Treatment	Other organs with metastasis	Time from primary lesion diagnosis (months)	Treatment	Living state	Survival time (months)
1	54/M	Rectum	AC	Surgery + chemotherapy	Bilateral	24	Found on follow-up	Surgery	Lung	19	Surgery	Alive	5
2	36/F	Ascending colon	AC	Chemotherapy	Left	4	Enlarging neck mass	Chemotherapy	Lung + liver	Synchronous (liver), 7 (lung)	Chemotherapy	Dead	5
3	45/M	Rectum	AC	Surgery + chemotherapy	Left	66	Found on follow-up	Surgery + chemotherapy	Lung + mediastinum + adrenal	40 (lung), 55 (adrenal)	Chemotherapy	Alive	27
4	44/F	Descending colon	AC	Surgery + chemotherapy	Right	28	Found on follow-up	Surgery + chemotherapy	Lung + liver	10 (ovary), 17 (lung)	Surgery + chemotherapy	Alive	5

Survival time is the time from thyroid metastasis from colorectal carcinoma to death or last follow-up

*AC* adenocarcinoma

**Table 2:** Seventeen cases of thyroid metastases from CRC, the previously reported cases in the literature

Authors	Age (year)/sex	Primary site	Histology	Treatment	Site of thyroid metastasis	Time from primary lesion diagnosis (months)	Clinical manifestation	Treatment	Other organs with metastasis	Time from primary lesion diagnosis (months)	Treatment	Living state	Survival time (months)
Nakamura et al. [1]	58/F	Sigmoid	AC	Surgery	Left	96	Found on follow-up	Surgery + chemotherapy	Lung	60	Surgery	Unknown	Unknown
Goatman et al. [3]	82/M	Rectum	AC	Surgery	Left	60	Found on follow-up	Surgery	Lung	60	Surgery	Unknown	Unknown
Poon et al. [4]	64/M	Ascending colon	AC	Surgery + chemotherapy	Multinodular	12	Dyspnea, dysphagia	Chemotherapy	Lung	14	Chemotherapy	Dead	18
Poon et al. [4]	53/F	Sigmoid	AC	Surgery + chemotherapy	Bilateral	Synchronous	Enlarging neck mass, dyspnea, dysphagia	Surgery + chemotherapy	Lung + liver	Synchronous	Untreated	Dead	10
Cheung et al. [5]	50/F	Rectum	AC	Surgery + chemotherapy	Right	64	Enlarging neck mass	Chemotherapy	Lung + liver	60	Surgery	Alive	Alive
Cheung et al. []	54/F	Rectum	AC	Surgery + chemotherapy	Left	60	Dry cough, hoarse voice, wheezing	Surgery + chemotherapy	Lung	12	Surgery + chemotherapy	Dead	42
Payandeh et al. [6]	54/M	Rectum	AC	Surgery	Multinodular	8	Dyspnea	Chemotherapy + Bev	Lung	Synchronous	Chemotherapy + Bev	Alive	Alive
Fujita et al. [7]	28/F	Rectum	AC	Surgery + chemotherapy	Right	Synchronous	Dysphagia	Surgery + chemotherapy	Lung	3	Untreated	Dead	6
Kim et al. [8]	68/F	Sigmoid	AC	Surgery	Right	24	Enlarging neck mass	Radiotherapy + supportive treatment	Lung	Synchronous	Supportive treatment	Unknown	Unknown
Boleas et al. [9]	80/F	Ascending colon	AC	Surgery + chemoradiotherapy	Bilateral	84	Dyspnea, wheezing	Surgery	Lung + bone + peritoneum	48	Surgery	Dead	12
Akimaru et al. [10]	67/M	Ascending colon	AC	Surgery	Left	72	Enlarging neck mass, hoarse voice	Chemotherapy	Lung + brain	72	Chemotherapy	Dead	4
Choufani et al. [11]	75/F	Sigmoid	AC	Surgery	Multinodular	84	Dyspnea	Surgery + radiotherapy	None	Unknown	Unknown	Dead	12
Takashima et al. [12]	82/M	Rectum	AC	Surgery	Left	24	Enlarging neck mass	Unknown	Unknown	Unknown	Unknown	Unknown	Unknown
Kameyama et al. [13]	82/M	Sigmoid	AC	Surgery	Right	24	Unknown	Unknown	Lung + liver + kidney + others	24	Unknown	Dead	12
Hacker et al. [14]	77/M	Rectum	AC	Surgery + chemoradiotherapy	Right	84	Enlarging neck mass	Surgery + chemotherapy	Lung	66	Surgery	Unknown	Unknown
Kumamoto et al. [15]	66/F	Ascending colon	AC	Surgery + chemotherapy	Left	42	Enlarging neck mass	Surgery + chemotherapy	Liver	30	Surgery	Alive	Alive
Mesko et al. [16]	59/F	Rectum	AC	Surgery + chemotherapy	Right	20	Found on follow-up	Surgery + supportive treatment	Bone + kidney	20	Supportive treatment	Unknown	Unknown

Survival time is the time from thyroid metastasis from colorectal carcinoma to death or last follow-up

*AC* adenocarcinoma

## Methods

A retrospective analysis was performed for four cases of thyroid metastases from CRC, treated in our center between January 2005 and December 2015 (Table 1). The patients with CRC in our center were monitored every 3 months for the first 2 years, and every 6 months thereafter, with routine follow-up examinations including medical history taking, physical examination, carcinoembryonic antigen assessment, and imaging examinations such as thoracic, abdominal, and pelvic computed tomography (CT) and/or magnetic resonance imaging (MRI). For patients with symptoms or signs noted during the physical examination of the thyroid and neck, thyroid function tests and cervical CT or ultrasonography were performed, followed by fine needle aspiration biopsy when deemed necessary.

In addition, the relevant literature was searched using PubMed, resulting in the identification of 17 patients with detailed information available [1, 3–16] (Table 2). The clinical data and follow-up information of our patients and the previously reported cases were collected and compared.

## Case histories

### Case 1

**Fig. 1:**
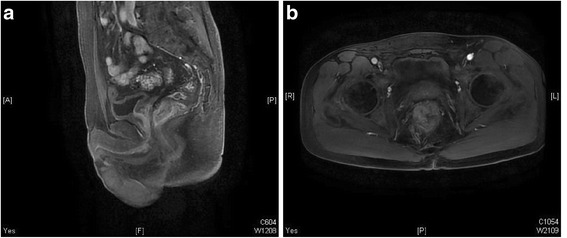
Primary lesion of case 1. The primary lesion was detected in the upper rectum on magnetic resonance imaging, which also revealed swollen pararectal lymph nodes. **a** Sagittal section; **b**: transverse section

**Fig. 2:**
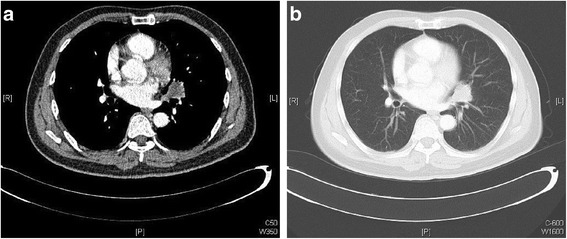
Lung metastases of case 1. Computed tomography scan of the lungs revealed multiple nodular lesions. **a** Mediastinal window; **b** pulmonary window

**Fig 3.**
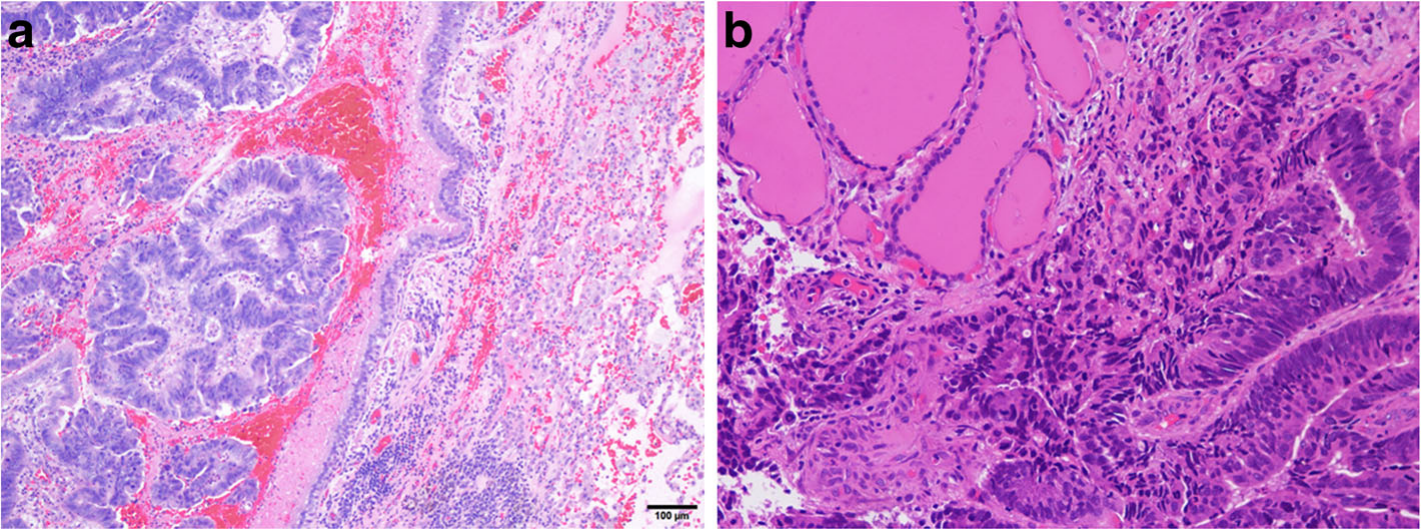
**a** Microphotograph of a lung metastasis in case 1 (hematoxylin and eosin stain, magnification ×10). **b** Microphotograph of a thyroid metastasis (hematoxylin and eosin stain, magnification ×20)

**Fig. 4:**
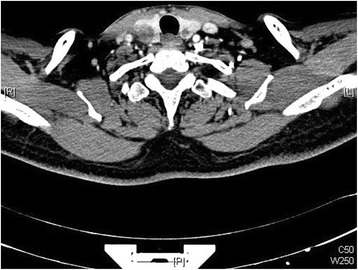
Thyroid metastases of case 1. Coronal computed tomography scan revealed bilateral solid nodules in the thyroid gland

On December 2013, a 54-year-old man presented with a 2-month history of hematochezia. A rectal adenocarcinoma, 4 cm from the anus, was detected on colonoscopy and diagnosed by biopsy. Rectal MRI revealed a malignant tumor (T3N1) (Fig. 1). No distant metastases were evident on CT scan of the thorax and abdomen. Neoadjuvant chemoradiotherapy (50 Gy/25 fractions, capecitabine 1500 mg bid) was administered. He received XELOX combination chemotherapy followed by chemoradiation, with an excellent response after 2 cycles. An abdominal perineal resection (Miles operation) was performed on March 2014. The pathologic stage after surgery was T3N0M0. Subsequently, he received 2 cycles of XELOX combination chemotherapy and 2 cycles of single-agent chemotherapy with capecitabine. On July 2015, he experienced recurrence with pulmonary metastases (Fig. 2) and underwent partial resection of the left lung (Fig. 3a). On December 2015, a coronal CT scan revealed bilateral solid nodules in the thyroid gland (Fig. 4). Thyroid metastases from CRC were confirmed by fine needle aspiration biopsy and histology results of the thyroid nodules. As a result, the patient underwent right lobectomy and partial left lobectomy of the thyroid gland on January 2016 (Fig. 3b). Unfortunately, he experienced recurrence with adrenal gland metastases on March 2016. Currently, the patient is undergoing preoperative FOLFIRI combination chemotherapy.

### Case 2

On December 2010, a 36-year-old woman presented with an enlarging neck mass. She was diagnosed with liver metastases from CRC, and an ascending colon adenocarcinoma was detected on colonoscopy and diagnosed by biopsy on August 2010. The patient underwent 6 cycles of XELOX combination chemotherapy from August 2010. Thyroid metastases from CRC were confirmed by fine needle aspiration biopsy and histology results of the left lobe thyroid nodules. The tumors showed wild-type *KRAS* status, and the patient consequently joined the experimental group of a randomized controlled, multi-center, prospective clinical study of a recombinant chimeric monoclonal anti-EGFR antibody combined with irinotecan. However, the patient’s condition was progressing after 3 cycles of treatment, and she hence quit the trial and instead underwent oral S-1 chemotherapy. On March 2011, a CT scan of the thorax revealed multiple bilateral lung metastases. Taking into account the fact that the patient did not tolerate combination chemotherapy, she chose to continue oral chemotherapy with S-1. Unfortunately, she died due to multiple organ failure on May 2011.

### Case 3

A 45-year-old man was diagnosed with stage IIIA rectal adenocarcinoma on August 2007, which was treated with anterior resection followed by 12 cycles of adjuvant XELOX combination chemotherapy. He experienced recurrence with pulmonary metastases on December 2010. Subsequently, he chose to undergo palliative therapy with 16 cycles of mFOLFOX6 combination chemotherapy.

On March 2012, a CT scan of the abdomen revealed right adrenal metastasis. The patient underwent gamma knife treatment of the right adrenal metastasis and lung metastases on April 2012 and September 2012, respectively. However, a thyroid metastasis from the rectal adenocarcinoma was found on February 2013. Thus, he received 3 cycles of XELOX combination chemotherapy followed by surgery. He remained well without any further treatment. On December 2015, an enlarging neck mass was found and thyroid metastases from CRC were confirmed by fine needle aspiration biopsy and histology results; however, the patient refused to accept the treatment and chose to instead be treated conservatively on regular follow-ups.

### Case 4

A 44-year-old woman was diagnosed with descending colon adenocarcinoma on August 2013, which was treated with left hemicolectomy. She received double oophorectomy because of right accessory metastasis on June 2014. She only underwent 2 cycles of FOLFIRI combination chemotherapy followed by surgery, because she did not tolerate combination chemotherapy. A left lung metastasis was detected on positron emission tomography-CT, and left lung wedge resection was performed on January 2015. Taking into account the fact that the patient did not tolerate combination chemotherapy, she chose to undergo oral chemotherapy with S-1 from February 2015. However, a right-lobe thyroid metastasis was found on routine follow-up examination, which was treated with right lobectomy and right neck dissection on December 2015. Thyroid metastasis from CRC was confirmed by postoperative pathology. Postoperatively, she received single-agent chemotherapy with capecitabine from January 2016, and she is currently well

## Results

The median age of the 21 patients was 59 years (44.5 and 66 years for our patients and the previously reported cases, respectively). Fifteen (71.4%) primary tumors were distributed throughout the distal colon or rectum (75% [3/4] in our center and 70.5% [12/17] in the previously reported cases). According to our analysis, we found that 81.0% of patients (17/21) showed concomitant lung metastasis. Among them, all four patients in our center showed lung metastasis, and 75% (3/4) developed thyroid metastases after lung metastasis. Among the previously reported cases, the corresponding proportions were 76.5 and 76.5% (13/17) of patients, respectively. The median time after primary tumor diagnosis to thyroid metastasis development was 28 months (26 months in our center and 35 months in the previously reported cases). One patient with advanced CRC in our center died 5 months after thyroid metastasis was identified, while the remaining three patients are currently alive (longest follow-up time, 27 months). The median survival time after thyroid metastasis during 3 years of follow-up of the previously reported 17 patients was 12 months. There was no difference in the overall survival between patients treated non-surgically (8/21) and patients undergoing thyroidectomy alone or thyroidectomy with adjuvant therapy (13/21) (*p =* 0.388). In addition, we found that the overall survival of the patients whose other metastases were treated with radical treatment was superior to that of those treated with palliative treatment (*p =* 0.022).

## Discussion

Thyroid metastasis of malignant tumors is observed in 1.9–9.5% of histologically examined autopsy cases [1], with thyroid metastases from non-thyroid malignancies remaining a rare occurrence in clinical practice, comprising only 1.4–3% of all thyroid neoplasms [2]. Studies from the USA and UK, including 15 cases of metastatic thyroid tumors, found that the kidney was the most common primary source of thyroid metastasis, while only one case showed thyroid metastasis from CRC [17]. Between January 2005 and December 2015, there were only four cases of thyroid metastasis from CRC in our center (Table 1), and we also collected relevant cases by a literature search using PubMed, which identified 17 patients with detailed information available (Table 2).

Willis [18] hypothesized that there were two possible reasons for why metastasis to the thyroid gland is so uncommon. One is a mechanical explanation, namely that the thyroid gland is an organ that receives an extremely abundant supply of arterial blood. This rapid blood flow rate prevents tumor cells from remaining fixed in the thyroid gland. The second is a chemical explanation, i.e., that the high oxygen saturation and high iodine content in the thyroid gland tissue may prevent the growth of tumor cells [18]. In fact, thyroid diseases that lead to reduced blood flow or low iodine levels, such as Hashimoto’s thyroiditis, have been suggested as potential risk factors for the development of thyroid metastases. However, in the reported patients with thyroid metastases from CRC, we found that the majority of patients were euthyroid and that the early symptoms and signs tended to be subtle. The clinical features, such as an enlarging neck mass, dyspnea, and dysphagia, appear during the course of disease progression. Three out of four patients (75%) in our center were diagnosed on detailed routine follow-up examination, as compared to only three out of the previously reported 17 cases. This may be related to our relatively regular follow-up schedule, indicating its contribution to the early detection and diagnosis of thyroid metastases from CRC in our clinical work.

In contrast to the much greater preponderance of primary thyroid tumors in women compared to that in men, the picture regarding thyroid metastases from CRC is less clear. Furthermore, a number of studies have reported that patients with thyroid metastasis from colon cancer tend to be older than 60 years [1, 3, 5, 19–22]. This may be related to the fact that most primary CRC patients are older at diagnosis or that older patients are more prone to organic or functional thyroid disorders, which may increase the risk of thyroid metastases from CRC. Herein, in the identified 21 patients, the female-to-male ratio was 1:1.2, and the median age of the 21 patients was 59 years. Among them, the median ages of our patients and previously reported cases were 44.5 years (range, 36–54 years) and 66 years (range, 28–82 years), respectively.

For patients with thyroid metastases from CRC, the primary tumor is mainly located in the distal colon or rectum. According to our statistics, 71.4% of the primary tumors (15/21 cases) were distributed throughout the distal colon or rectum (75% [3/4 cases] in our center and 70.5% [12/17 cases] in the previously reported cases in Table 2). Thyroid metastases from CRC usually occur in patients with advanced CRC and often occur concomitant with, or appear after, other metastases [23]. Herein, we found that all four patients treated in our center showed combined pulmonary and other organ metastases, including liver, ovarian, and adrenal gland metastases. In the previously reported patients [1, 3–16] (Table 2), 76.5% (13/17), 23.5% (4/17), and 23.5% (4/17) showed concomitant pulmonary, liver, and other organ metastases except to the lungs and liver, respectively.

The diagnosis of thyroid metastasis is frequently delayed because the early symptoms and signs are subtle, and symptomatic thyroid dysfunction is rare. The time from CRC diagnosis to thyroid metastasis varies. Some tumors are metachronous while others are synchronous. In the present study, the median time from the primary CRC diagnosis to thyroid metastasis was 28 months in all patients (26 months in our center and 35 months in the previously reported cases in Table 2). Hematogenous spread is the most important pathway for metastasis [1], because, in many cases, thyroid metastasis is accompanied by liver and, especially, lung metastases [1, 5, 6, 8, 9, 14]. Furthermore, as described above, 81.0% of all patients (17/21) showed concomitant lung metastasis. Among them, all four patients in our center showed lung metastasis, and 75% (3/4) developed thyroid metastasis after lung metastasis. In the previously reported cases [1, 3–10, 13, 14], the corresponding proportions were 76.5% (13/17) and 76.9% (10/13) of patients, respectively. Therefore, CRC can be considered to metastasize to the thyroid gland mainly via the hematogenous pathway, i.e., CRC spreads to the thyroid gland through the portal vein, vena cava, and pulmonary vein [2–7]. However, it has also been proposed that the special physiology and pathology of the vertebral venous system enable such tumors to bypass the portal vein, pulmonary vein, and vena cava and to be transferred directly to the thyroid or any part of the body, without entering the thoracic and abdominal cavity [24]. As summarized in Table 2, a patient had no other organ metastasis except for thyroid metastasis, while another three of the previously reported patients and one patient treated in our center developed thyroid metastasis before lung metastasis; these findings support the existence of a vertebral venous system for metastatic spread.

The most common clinical features of thyroid metastases from CRC include an enlarging neck mass, dyspnea, dysphagia, dry cough, hoarse voice, and wheezing, among others. One patient treated in our center had an enlarging neck mass at the time of presentation, while neck masses were found in the other three patients on detailed routine follow-up examination. As seen in Table 2, there were seven (41.2%), five (29.4%), and three (17.6%) cases with an enlarging neck mass, dyspnea, and dysphagia, respectively, out of the previously reported cases, and only three patients (17.6%) were found on routine follow-up examination. The cases in our center were euthyroid, and the early symptoms and signs were subtle when they were diagnosed. This finding may be related to our regular and detailed follow-up and indicates its contribution to both the early detection and diagnosis of thyroid metastases from CRC in our clinical work and, potentially, to the reduced prevalence of acute symptoms such as dyspnea and dysphagia.

In many studies from overseas, most patients were reportedly asymptomatic early in the disease course [1, 3, 16], and some patients with thyroid metastases from CRC showed no clinical features until death, which may be the reason for why the proportion of histologically diagnosed autopsy cases is higher than that of cases diagnosed in clinical practice [2, 5, 15]. Currently, the routine follow-up examinations of patients with CRC include only thoracic, abdominal, and pelvic examinations, and not neck evaluations, which may lead to misdiagnosis or delayed diagnosis of thyroid metastasis. A previous clinical study found that 10 to 15% of patients with colorectal cancer would have lung metastases [25]. According to our statistics, there are 13,000 patients with colorectal cancer treated in our center between January 2005 and December 2015, of which 1560 patients undergo lung metastasis, which accounting for 12%. All four cases of thyroid metastases from CRC treated in our center showed combined pulmonary metastases, accounting for 0.26%. For the patients with thyroid metastases of CRC, the proportion of combined pulmonary metastases is quite high. In our study, 81.0% of all patients (17/21) showed concomitant lung metastasis. Therefore, the presurgical evaluation and postoperative follow-up should include the thyroid, especially in high-risk patients with CRC lung metastases and/or a history of thyroid nodules. The diagnosis of thyroid metastases from CRC should be made by a combination of history taking, thyroid function tests, and imaging studies. If required, fine needle aspiration biopsy and pathological examination for suspicious patients, along with immunohistochemical analyses, should be performed to confirm the diagnosis.

As mentioned above, thyroid metastasis from CRC is a manifestation of advanced CRC. Patients with advanced CRC generally undergo comprehensive treatment with chemotherapy. Furthermore, for patients with thyroid metastases from CRC, aggressive surgical treatment can help avoid the appearance of crises such as dyspnea and dysphagia, and may thus result in a better prognosis and quality of life of the patients [4, 5, 23]. The chemotherapy regimens used include combination chemotherapy with XELOX, mFOLFOX6, or FOLFIRI [1, 4, 5]. Accordingly, for patients suitable for targeted therapy, combination chemotherapies including bevacizumab or cetuximab should be considered [6].

Thyroid metastases from CRC are associated with a poor prognosis and high mortality. Except for the patient described in case 4 herein, who died 5 months after the diagnosis of thyroid metastasis from CRC, the other patients are currently alive, with the longest follow-up being 27 months. The median survival time after thyroid metastasis during 3 years of follow-up of the previously reported 17 patients was 12 months (Table 2). A number of studies, as well as our present study, have shown that the majority of patients with thyroid metastases from CRC usually have concomitant metastases in other organs and a poor prognosis [1, 7, 12, 13]. Hence, it is obvious that thyroid metastases represent a CRC end-stage manifestation. The prognosis of patients with thyroid metastases from CRC are related to many factors, including the grade of malignancy of the primary lesion, the presence of other metastases, and whether the metastases are timely diagnosed and a radical treatment strategy is employed for the thyroid lesions [1, 26–28]. A shorter mean survival in patients who were treated non-surgically (25 months), as compared to in patients who underwent thyroidectomy alone or thyroidectomy with adjuvant therapy (34 months), was reported in one series [24]. However, according to our analysis, the overall survival in patients treated non-surgically (8/21) and patients who underwent thyroidectomy alone or thyroidectomy with adjuvant therapy (13/21) did not significantly differ (*p =* 0.388). In addition, we found that the overall survival of patients whose other metastases were treated with radical surgery was superior to those undergoing palliative treatment (*p =* 0.022). These results indicate that while there is no significant relationship between thyroidectomy and overall survival, radical treatment of the other metastases influences the survival of the patients. Aggressive surgical treatment can moreover help avoid the appearance of crises such as dyspnea and dysphagia and thus enhance the patients’ quality of life.

## Conclusions

Thyroid metastases from CRC are rare in clinical practice and are a manifestation of advanced CRC. Attention should be paid to ensure timely diagnosis of thyroid metastases from CRC, and aggressive treatment of these tumors should be performed in order to prolong the survival, enhance the quality of life, and improve the prognosis of these patients.

## Abbreviations

CRC: Colorectal cancer
CT: Computed tomography
MRI: Magnetic resonance imaging
